# Voriconazole-Induced Squamous Cell Carcinoma after Hematopoietic Stem Cell Transplantation Showing Early-Stage Vascular Invasion

**DOI:** 10.3390/dermatopathology7030008

**Published:** 2020-10-09

**Authors:** Yumi Sawada, Yasuo Nakai, Naho Yokota, Koji Habe, Akinobu Hayashi, Keiichi Yamanaka

**Affiliations:** 1 Department of Dermatology, Mie University, Graduate School of Medicine, Tsu, Mie 514-8507, Japan; y.swd22@gmail.com (Y.S.); nakai-y@clin.medic.mie-u.ac.jp (Y.N.); moco0503@icloud.com (N.Y.); habe-k@clin.medic.mie-u.ac.jp (K.H.); 2 Department of Pathology, Mie University, Graduate School of Medicine, Tsu, Mie 514-8507, Japan; ahayashi@med.mie-u.ac.jp

**Keywords:** hematopoietic stem cell transplantation, immunosuppressive therapy, squamous cell carcinoma, vascular invasion, voriconazole

## Abstract

Voriconazole is a triazole antifungal agent used for the prevention and treatment of fungal infections in immunocompromised patients. Prolonged voriconazole therapy may induce phototoxicity and lead to the development of malignant neoplasms of the epidermis, such as squamous cell carcinoma (SCC), especially in immunocompromised patients. We report a case of voriconazole-induced phototoxicity and SCC occurring after hematopoietic stem cell transplantation (HSCT) in a 56-year-old man with primary myelofibrosis. The patient developed chronic graft-versus-host disease (GVHD) post-transplantation and had been receiving long-term immunosuppressive treatment. A year after the initiation of voriconazole therapy for prophylaxis, he developed keratotic erythema, followed by SCC with vascular invasion after three years. A review of SCC in HSCT recipients suggests that the prolonged use of voriconazole is regarded as a risk for SCC after HSCT in patients with chronic GVHD on immunosuppressive therapy. Moreover, a histological examination of the completely resected tumor revealed vascular invasion in this case, although neither the clinical features nor the histological findings of the preoperative biopsy suggested invasive carcinoma. This case may partially explain why voriconazole-associated SCCs show a more aggressive clinical course than non-voriconazole SCCs do.

## 1. Introduction

Voriconazole is a triazole antifungal agent that is often used not only therapeutically, but also prophylactically, especially in immunocompromised patients. Long-term use of voriconazole has been reported to induce phototoxicity and even squamous cell carcinoma (SCC) [1,2,3,4,5]. The risk of SCC after hematopoietic stem cell transplantation (HSCT) has also been reported, indicating the involvement of chronic graft-versus-host disease (GVHD) and immunosuppressive treatment for GVHD [1,2,6,7,8]. We report a case of multiple actinic keratosis and invasive SCC in a patient on voriconazole and immunosuppressive therapy after HSCT and chronic GVHD.

## 2. Case Report

A 56-year-old man was diagnosed with primary myelofibrosis five years ago and had received an allogeneic HSCT from his sister. He underwent immunosuppressive therapy with tacrolimus along with fluconazole therapy for prophylaxis. Three months later, he developed a hepatic disorder accompanied with thrombocytopenia, suggesting chronic GVHD; therefore, immunosuppressive therapy was continued. About one year after the HSCT, he developed late-onset noninfectious pulmonary complications triggered by an infection treated with corticosteroids and antibiotics. Additionally, fluconazole was replaced by 400 mg/day of voriconazole (Vfend^®^, Pfizer, Tokyo, Japan) and maintained for long-term prophylaxis. One year after initiating voriconazole therapy, an infiltrating erythema appeared on the face and left arm. Chronic GVHD was suspected and treated with topical corticosteroids. During the course of treatment with voriconazole, he had persistent erythema on the head and face that was unresponsive to topical corticosteroids, and new multiple erosive and hyperkeratotic lesions appeared. He was subsequently referred to our department.

**Figure 1:**
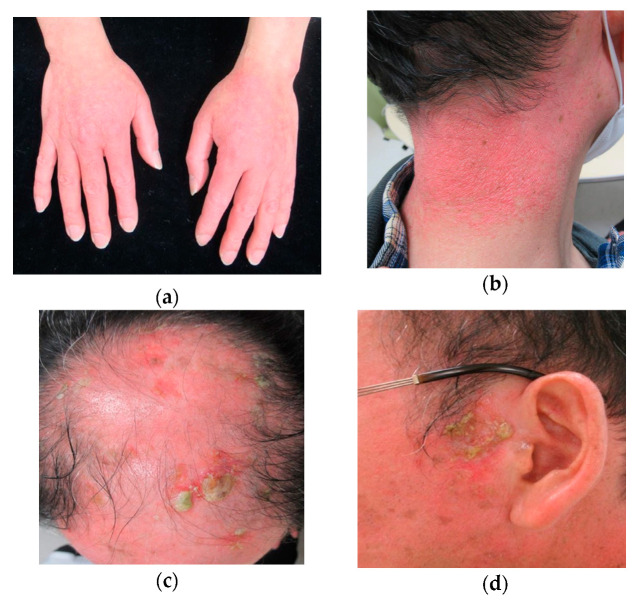
Clinical features of the skin lesions of the patient. (**a**,**b**) Photo-distributed erythema on sun-exposed skin areas; (**c**) multiple erosive and hyperkeratotic lesions on the scalp; (**d**) hyperkeratotic lesion on the left preauricular area.

The physical examination revealed a non-pruritic, non-tender photo-distributed erythema on the sun-exposed areas of the skin (head, face, posterior neck, arms, and the back of the hand) and keratotic plaques and erosions, particularly on the scalp and left preauricular area (Figure 1). No sclerotic skin changes typical of GVHD were present.

**Figure 2:**
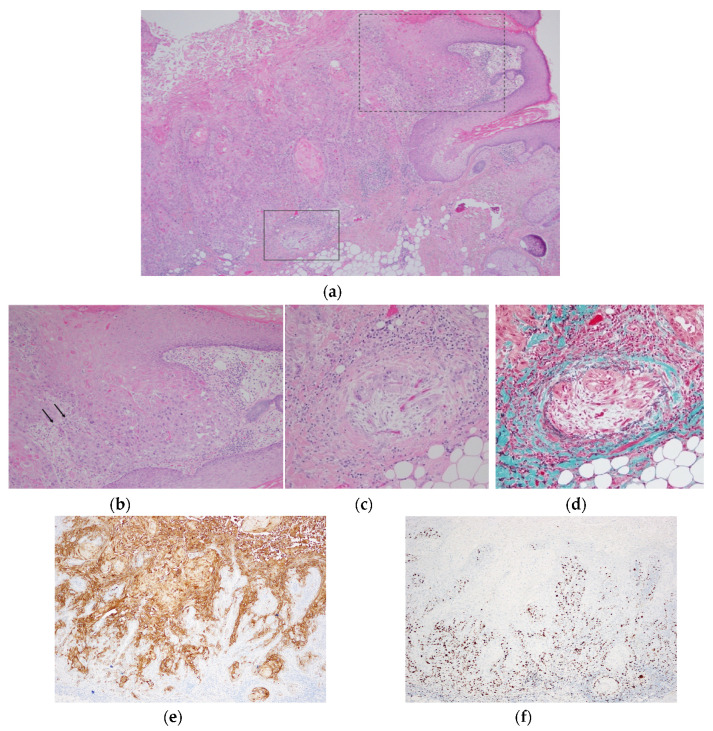
Histopathologic examination of the left preauricular lesion. (**a**) Invasive squamous cell carcinoma (SCC) arising from the epidermis is shown (Hematoxylin-Eosin staining (HE), ×40). Solar elastosis is also seen in the dermis at the lower right of the figure. The dotted square indicates where the origin of the SCC from the overlying epidermis is, and the solid square indicates where the vascular invasion is; (**b**) magnified image of the dotted square lesion. The arrows indicate the site of invasion (HE, ×100); (**c**) higher-magnification image shows the vascular invasion (HE, ×200); (**d**) the elastic lamina of the vein is highlighted (Elastica-Masson staining, ×200); (**e**) cytokeratin 17 expression was observed in the epithelium (×40); (**f**) increased expression of Ki-67 in the epithelium is also observed (×40).

Biopsies from the hyperkeratotic lesions on the scalp and preauricular area revealed actinic keratosis (AK). Voriconazole was then discontinued and replaced by fluconazole. Three lesions on the scalp and another lesion on the left preauricular area were surgically resected. Although the biopsy results revealed AK, the biopsied lesion on the scalp was excised with a 6 mm surgical margin along with the underlying periosteum. The other lesions on the scalp were resected with a 4 mm margin on the epicranial aponeurosis, and a 4 mm margin including subcutaneous fat for the preauricular lesion. On histopathological examination, the biopsied lesions on the scalp and preauricular were diagnosed as SCC with vascular invasion, and one of the non-biopsied lesions was diagnosed as SCC, and the other was AK (Figure 2). Thereafter, the patient received postoperative radiation therapy. No recurrence or metastasis has occurred to date even six months postoperatively.

## 3. Discussion

Voriconazole is a broad-spectrum triazole antifungal agent for the treatment of invasive fungal infections, particularly for the *Aspergillus*, *Candida*, *Fusarium*, and *Scedosporium* species. It is commonly used not only for treatment, but also for prophylaxis because of its broad antifungal spectrum, oral bioavailability, and generally well-tolerated side-effect profile [1,2]. However, prolonged voriconazole therapy may induce phototoxicity, including photosensitivity, and an increased risk of SCC, particularly in immunocompromised patients. The mechanism is not well understood, but previous studies have attributed it to the direct phototoxicity by voriconazole or its metabolite, voriconazole N-oxide, or its indirect retinoid-like mechanism [2,3,4,9,10]. Voriconazole-induced photosensitivity presents as a sunburn response on the sun-exposed areas, including erythema, cheilitis, exfoliative dermatitis, pseudoporphyria, and discoid lupus erythematosus. Generally, such reactions are reversible upon discontinuation of the drug, and disappear in about four weeks [2,3,11], but long-term use induces phototoxicity and development of malignant neoplasms of the epidermis, including AK and SCC, and even malignant melanoma [1,2,3,4,5,12]. The skin lesions caused by voriconazole go through a multistep process: erythema of the photo-exposed area lasting several months to one year, followed by AK in two–three years, and SCC in three–four years [5].

Previous case–control studies conducted in Europe and America indicate that patients who have undergone HSCT are at an increased risk of secondary malignancy, particularly SCC [1,2,6,7,8]. One reason for this is the association between chronic GVHD and prolonged immunosuppressive therapy. There are many theories about the etiological factors of SCC, and suppression of immunity is one such factor. There is a strong association between the risk of SCC and the duration of immunosuppressive drug use [2,6,7,8]. In patients undergoing HSCT, immunosuppressive therapy is required for more than six months unless they develop GVHD. In other words, the development and severity of GVHD may be strongly related to the duration of treatment with immunosuppressive medications. Furthermore, we found lower natural killer cell activity in the current case (1.0% and 2.3% at effector-to-target (E/T) ratios of 10:1 and 20:1, respectively; normal range 8.9–29.5% and 17.1–48.7%, respectively). The NK cell activity is reduced by various factors, such as aging, lifestyle, malignancy, immunodeficiency, and drugs [13], and in this case, immunosuppressive therapy for chronic GVHD was presumed to be the main factor. More importantly, the decrease in NK cell activity means reduced innate immunity, especially against cancer, thus leading to SCC. In summary, the development and severity of GVHD and use of immunosuppressive medications were suggested to be the major risk factors for the development of SCC after HSCT.

However, there are a limited number of reports in Japan on SCC in post-HSCT patients. This may be because of the difference in the incidence of SCC and chronic GVHD between ethnic groups [8]. In Japan, 10 cases of SCC in post-HSCT patients were reported, and among them, 5 patients developed chronic GVHD and the other cases of uncomplicated chronic GVHD were treated with voriconazole. Although the general risk factors for SCC (age, race, smoking, long-term ultraviolet (UV) light exposure, etc.) were also considered, the fact might suggest the involvement of voriconazole in the development of SCC. As noted earlier, voriconazole is often administered to immunocompromised patients, such as for prophylaxis in HSCT recipients, and is usually indicated for long-term use. This may increase the risk of developing SCC post-HSCT. In this case, the patient was administered immunosuppressive therapy for chronic GVHD along with voriconazole therapy for over two years. Both these factors may have contributed to the development of SCC. Additionally, the hyperkeratotic lesions that were preoperatively diagnosed as AK via biopsy proved to be vascular invasive SCC after histological examination of the completely resected tumor. Although voriconazole-associated SCC is reported to be more aggressive than non-voriconazole SCC [2,4,14], no study has probably reported a histologically proven vascular invasion in voriconazole-induced SCC. The findings of the previous reports and this case suggest the possibility that vascular invasion may develop even in the early stages of voriconazole-associated SCC. Thus, we would like to emphasize that when hyperkeratotic lesions are present in patients on voriconazole therapy, especially those who have undergone HSCT, the possibility of invasive cancer on histopathology should be strongly considered, even if the clinical findings or preoperative diagnosis via biopsy suggest neoplasm in situ.

Although there are several reports on voriconazole-induced phototoxicity and carcinogenicity, the mechanism has not been elucidated so far, and there is a possibility that physicians will continue to administer voriconazole without being aware of its side effects. Particularly in post-transplant patients, it is often difficult to distinguish voriconazole-induced lesions from those of GVHD, and the eruption may be treated as a part of GVHD [15]. Since the phototoxic symptoms caused by voriconazole can be ameliorated by early detection and discontinuation of the drug, healthcare providers, especially dermatologists, should be fully aware of this side effect. Moreover, voriconazole therapy may increase the risk of developing SCC with vascular invasion under immunosuppression after HSCT or along with the complications of GVHD; thus, its use should be carefully followed up. It is also important to create patient awareness regarding these side effects and instruct them about the benefits of protection against UV radiation.
